# Editorial: Novel Plant Molecules Regulating the Interaction With Pathogenic and Beneficial Fungi

**DOI:** 10.3389/fpls.2021.644546

**Published:** 2021-01-27

**Authors:** Ivan Baccelli, Laura Bertini, Richard Hickman, Antonio Leon-Reyes, Silvia Proietti

**Affiliations:** ^1^Institute for Sustainable Plant Protection, National Research Council of Italy, Florence, Italy; ^2^Department of Ecological and Biological Sciences, University of Tuscia, Viterbo, Italy; ^3^Plant-Microbe Interactions, Department of Biology, Utrecht University, Utrecht, Netherlands; ^4^Laboratorio de Biotecnología Agrícola y de Alimentos-Ingeniería en Agronomía, Colegio de Ciencias e Ingenierías El Politécnico, Universidad San Francisco de Quito USFQ, Quito, Ecuador

**Keywords:** molecular plant-microbe interaction, microbiome, endophytes, mycorrhizae, plant diseases, plant evolution, plant immunity, stress resistance

Plants started colonizing land ~450–470 million years ago (Humphreys et al., 2010; Rubinstein et al., 2010; Parfrey et al., 2011). With remarkable coincidence, the first symbiotic interaction between fungi and plants can be dated back to 400–460 million years ago (Remy et al., 1994; Redecker et al., 2000).

In 1975, Pirozynski and Malloch hypothesized that colonization of land by plants was possible only because a symbiotic association between a “semi-aquatic ancestral alga” and a “mycorrhizal partner” (referred to as an “aquatic fungus” by the authors) occurred. For the authors, terrestrial plants are “the product of this ancient and continuing partnership” which has allowed plants “to cope with the problems of desiccation and starvation associated with terrestrial existence” (Pirozynski and Malloch, 1975). Moreover, “land plants never had any independence” (from mutualistic symbiosis), and “if they had, they could never have colonized the land.” According to recent data, arbuscular mycorrhizal fungi actually appeared as drivers of plant terrestrialization in early Palaeozoic land ecosystems (Humphreys et al., 2010; Field et al., 2012; Loron et al., 2019).

Coevolution between plants and fungi has obviously led both partners to evolve mechanisms of interaction that nowadays appears extremely sophisticated. Plants can produce molecules able to promote beneficial interactions and molecules able to counteract pathogenic interactions. They can sense pathogens and trigger the activation of defenses as well as communicate with beneficial fungi and modify the plant transcriptome and metabolome in order to accommodate symbiotic associations. The outcome of the interaction may have implications in terms of plant growth, development, and stress resistance.

In this issue we will highlight discoveries in the field of plant-fungi interactions, particularly around the role of plant molecules, with the belief that the development of novel plant protection strategies will be greatly assisted in the future by understanding the mechanisms of plant-microbe communication.

## Pathogenic Plant-Fungi Interactions

Plant molecules may act as either resistance or susceptibility factors during the plant-pathogen interaction, and their role may also go beyond this and affect plant growth and developmental traits or tolerance to abiotic stresses. Their identification and characterization is thus essential to develop sustainable control strategies for plant protection.

Wild tomato species are a valuable source of resistance to powdery mildew caused by *Oidium neolycopersici*. This is the case of *Solanum habrochaites* G1.1560 which carries the resistance gene *Ol-1*. Zhang Y. et al. identified a new gene required for *Ol-1-*mediated resistance. The gene, named ShORR-1 (*Solanum habrochaites Oidium* Resistance Required-1) was shown to encode a membrane-localized protein. By overexpression and silencing studies, it was demonstrated that ShORR-1 plays a role in resistance in *S. habrochaites* G1.1560. However, a ShORR-1 homolog differing in 13 aa residues from the susceptible tomato cultivar Moneymaker was shown to confer instead pathogen susceptibility, revealing how gene variants may differently turn the interaction into resistance or susceptibility.

A Type I Rac/Rop GTPase named TaRac6was studied in *Triticum aestivum* by Zhang Q. et al. *Puccinia striiformis* f. sp. *tritici* isolates CYR23 (leading to incompatible interaction, i.e., resistance) and CYR31 (leading to compatible interaction, i.e., disease) were used to investigate the role of TaRac6. Transient expression of TaRac6 inhibited Bax-triggered plant cell death in *N. benthamiana*. In addition, the gene was up-regulated 24 h after infection only in the compatible interaction. Importantly, silencing of TaRac6 by virus induced gene silencing (VIGS) led to higher production of hydrogen peroxide and enhanced resistance to *P. striiformis* f. sp. *tritici* CYR31, suggesting that TaRac6 functions as a susceptibility factor.

Membrane-bound transcription factors (MTFs) belonging to the basic leucine zipper (bZIP) family act as key components of stress signaling pathways in endoplasmic reticulum (ER). Wang et al. revealed how mRNA encoding a bZIP MTF in wheat, named TabZIP74, may undergo splicing and encode a new protein lacking the transmembrane domain which is mobilized to the nucleus. Knocking down TabZIP74 by VIGS enhanced wheat seedling susceptibility to *P. striiformis* f. sp. *tritici*, and decreased both drought tolerance and lateral root formation, demonstrating that TabZIP74 mRNA is induced to splice during biotic and abiotic stresses and acts as a positive regulator of wheat stripe rust resistance and drought tolerance, being also implied in root development.

Invertases irreversibly catalyze the cleavage of sucrose into glucose and fructose, exerting a pivotal role in carbon utilization and distribution, as well as immune responses to pathogens. Their activities are determined by proteinaceous inhibitors named C/VIFs (cell wall/vacuolar inhibitor of β-fructosidases). Su et al. characterized two putative invertase inhibitors from *Populus trichocarpa* named PtC/VIF1 and PtC/VIF 2, and showed that the two encoding genes were down-regulated in poplar roots during *Fusarium solani* infection, suggesting that invertase inhibitors may be involved in a sucrose-mediated defense pathway.

Pastor-Fernández et al. reported on the protective effects of two signaling peptides, systemin, and hydroxyproline-rich systemins (HypSys) from tomato, on *Arabidopsis thaliana*. The peptides were able to induce resistance against *Plectosphaerella cucumerina* infection indicating that *Arabidopsis* plants can sense peptides from phylogenetically distant plant species. In addition, it emerged how resistance was dependent on jasmonic acid signaling and led to enhanced PAMP-triggered immunity (PTI) responses upon infection.

Trdá et al. shed new light on a group of plant molecules named saponins, demonstrating that besides antifungal activity they may also possess resistance inducing ability. In particular, the terpenoid saponin aescin was able to induce resistance in *Brassica napus* against the fungus *Leptosphaeria maculans* by activating the SA pathway and oxidative burst. In *A. thaliana*, aescin induced SA-dependent resistance to the bacterium *Pseudomonas syringae* pv *tomato* DC3000.

Serine protease inhibitors (PIs) belonging to the Kunitz-type (PKPI) and Potato type I (Pin1) families are known to possess insecticidal and nematicidal activity. Turrà et al. investigated the ability of PKPI and Pin1 proteins to limit fungal and bacterial infection and influence plant growth. Transgenic *Nicotiana benthamiana* plants transiently expressing PKPI and Pin1 proteins turned out to be more resistant to *Botrytis cinerea, Alternaria alternata* and *Pseudomonas syringae* pv. *tabaci* infections. Systemic expression of these proteins resulted in plants with enhanced shoot and root biomass, revealing that members of PKPI and Pin1 family proteins can influence cell development, differentiation, and disease resistance to fungal and bacterial pathogens.

Finally, a new effector named AGLIP1, able to induce cell death in *Nicotiana benthamiana* and to suppress PAMP-triggered immunity in transgenic *Arabidopsis* lines was identified from *Rhizoctonia solani* by Li et al.

## Beneficial Plant-Fungi Interactions

Plant-associated fungi can bestow important benefits upon host plants. Nevertheless, plants need to manage the microbes residing inside them or surrounding their roots, i.e., the so-called microbiome. Among the strategies to achieve this, plants can produce exudates and other molecules that are able to shape root-associated microbial communities.

In their comprehensive review, Pascale et al. summarized the current knowledge on plant-microbiome interactions. More specifically, they described the mechanisms by which plants select their microbiome (via structural/chemical components) and presented well-characterized examples of microbiome recruitment by plants. Finally, they suggested approaches to exploit plant microbiomes and design synthetic communities that can be used to boost plant health and growth in a sustainable and reproducible manner.

Some of the molecules employed by plants to communicate with surrounding microorganisms originate from carotenoid precursors by oxidative cleavage, yielding a range of compounds known as apocarotenoids. Apocarotenoids are emerging as key regulators of plant–microbe interactions, in particular of the arbuscular mycorrhizal (AM) symbiosis: abscisic acid (ABA), strigolactones, blumenols, mycorradicins, and zaxinone play roles during different stages of the colonization process by AM fungi, as reviewed by Fiorilli et al.

Finally, in a context of increasing demand for animal-friendly endophytes harboring deterrent and insecticidal properties for the market of artificially infected grass cultivars (Johnson et al., 2013), Cagnano et al. performed a large-scale screening of *Epichloë* endophytes infecting *Schedonorus pratensis* and other forage grasses and investigated genetic diversity, geographic variation, and loline alkaloid levels.

## Author Contributions

The authors jointly defined the content of this Research Topic and all participated in the editing process. All authors made substantial, direct, intellectual contribution to the composing of this editorial and approved it for publication.

## Conflict of Interest

The authors declare that the research was conducted in the absence of any commercial or financial relationships that could be construed as a potential conflict of interest.

## References

[B1] Field K. J. Cameron D. D. Leake J. R. Tille S. Bidartondo M. I. Beerling D. J. (2012). Contrasting arbuscular mycorrhizal responses of vascular and non-vascular plants to a simulated Palaeozoic CO_2_ decline. Nat. Commun. 3:835. 10.1038/ncomms1831 22588297

[B2] Humphreys C. P. Franks P. J. Rees M. Bidartondo M. I. Leake J. R. Beerling D. J. (2010). Mutualistic mycorrhiza-like symbiosis in the most ancient group of land plants. Nat. Commun. 1:103. 10.1038/ncomms1105 21045821

[B3] Johnson L. J. De Bonth A. C. M. Briggs L. R. Caradus J. R. Finch S. C. Fleetwood D. J. (2013). The exploitation of epichloae endophytes for agricultural benefit. Fungal Divers. 60, 171–188. 10.1007/s13225-013-0239-4

[B4] Loron C. C. François C. Rainbird R. H. Turner E. C. Borensztajn S. Javaux E. J. (2019). Early fungi from the Proterozoic era in Arctic Canada. Nature. 570, 232–235. 10.1038/s41586-019-1217-0 31118507

[B5] Parfrey L. W. Lahr D. J. Knoll A. H. Katz L. A. (2011). Estimating the timing of early eukaryotic diversification with multigene molecular clocks. Proc. Natl. Acad. Sci. U.S.A. 108, 13624–13629. 10.1073/pnas.1110633108 21810989 PMC3158185

[B6] Pirozynski K. A. Malloch D. W. (1975). The origin of land plants: a matter of mycotrophism. Biosystems 6, 153–164. 10.1016/0303-2647(75)90023-4 1120179

[B7] Redecker D. Kodner R. Graham L. E. (2000). Glomalean fungi from the Ordovician. Science 289, 1920–1921. 10.1126/science.289.5486.1920 10988069

[B8] Remy W. Taylor T. N. Hass H. Kerp H. (1994). Four hundred-million-year-old vesicular arbuscular mycorrhizae. Proc. Natl. Acad. Sci. U.S.A. 91, 11841–11843. 10.1073/pnas.91.25.11841 11607500 PMC45331

[B9] Rubinstein C. V. Gerrienne P. de la Puente G. S. Astini R. A. Steemans P. (2010). Early Middle Ordovician evidence for land plants in Argentina (eastern Gondwana). New Phytol. 188, 365–369. 10.1111/j.1469-8137.2010.03433.x 20731783

